# Ecological comparison of cellular stress responses among populations – normalizing RT-qPCR values to investigate differential environmental adaptations

**DOI:** 10.1186/1472-6785-13-21

**Published:** 2013-05-16

**Authors:** Stefan Koenigstein, Kevin Pöhlmann, Christoph Held, Doris Abele

**Affiliations:** 1 Alfred-Wegener-Institute for Polar and Marine Research, Am Handelshafen 12, Bremerhaven D-27515, Germany

**Keywords:** Ecological physiology, Cellular stress response, Hsp70, RT-qPCR, Gene expression, Normalization, Intertidal, Nacella

## Abstract

**Background:**

Rising temperatures and other environmental factors influenced by global climate change can cause increased physiological stress for many species and lead to range shifts or regional population extinctions. To advance the understanding of species’ response to change and establish links between individual and ecosystem adaptations, physiological reactions have to be compared between populations living in different environments. Although changes in expression of stress genes are relatively easy to quantify, methods for reliable comparison of the data remain a contentious issue. Using normalization algorithms and further methodological considerations, we compare cellular stress response gene expression levels measured by RT-qPCR after air exposure experiments among different subpopulations of three species of the intertidal limpet *Nacella.*

**Results:**

Reference gene assessment algorithms reveal that stable reference genes can differ among investigated populations and / or treatment groups. Normalized expression values point to differential defense strategies to air exposure in the investigated populations, which either employ a pronounced cellular stress response in the inducible Hsp70 forms, or exhibit a comparatively high constitutive expression of Hsps (heat shock proteins) while showing only little response in terms of Hsp induction.

**Conclusions:**

This study serves as a case study to explore the methodological prerequisites of physiological stress response comparisons among ecologically and phylogenetically different organisms. To improve the reliability of gene expression data and compare the stress responses of subpopulations under potential genetic divergence, reference gene stability algorithms are valuable and necessary tools. As the Hsp70 isoforms have been shown to play different roles in the acute stress responses and increased constitutive defenses of populations in their different habitats, these comparative studies can yield insight into physiological strategies of adaptation to environmental stress and provide hints for the prudent use of the cellular stress response as a biomarker to study environmental stress and stress adaptation of populations under changing environmental conditions.

## Background

Increased air and water temperatures are recorded as a consequence of climate change, challenging the tolerance of ectothermal organisms by inducing increased physiological stress [1,2]. Modern ecophysiological approaches aim to integrate physiological and phylogenetic data to increase our understanding of how inter-individual variation in physiological responses to the environmental parameters shapes population responses to climate change [3,4]. Physiological data can provide the mechanistic link between environmental change and ecological success of species, and the comparison of different populations or closely related species on environmental gradients allows us to study adaptive responses and determine the respective roles of phenotypic plasticity and genetic adaptation, to model and predict responses to environmental change and to develop conservation strategies [5].

Gene expression data can provide a quantification of the physiological stress suffered by individual organisms, but to compare stress levels among populations, a common baseline has to be established. Real-time RT-qPCR is a fast, comparatively inexpensive and highly accurate method of gene expression quantification over a wide range of transcription intensities [6]. However, for correct quantification and comparability of gene expression data, RT-qPCR values need to be normalized to a set of suitable reference genes. While in a traditional approach, a single ‘housekeeping’ gene was used which was assumed to be universally stable, methodological advances in biomedical studies have demonstrated the necessity to characterize several candidate reference genes with respect to stability of their expression under the experimental conditions [7,8]. Choosing an inadequate reference gene can not only corrupt reliability of the data but, moreover, lead to erroneous conclusions, if the selected reference gene itself is regulated under the investigated conditions [9,10]. When two quantified genes are co-regulated, e.g. when they are part of the same metabolic path, the similarity of expression patterns can incorrectly point to high stability under the experimental setup. In an ecological context, in habitats with periodically changing conditions like the intertidal, temporal splitting of basic physiological functions and differential regulation of genes can be regularly expected, posing a potential problem for reference gene stability [11]. Furthermore, in studies of non-model organisms, less sequence data are available and the number of quantifiable genes is typically lower.

In order to overcome these limitations and improve the utility of real-time RT-qPCR gene expression studies for ecological comparisons, we suggest to apply the following methodological considerations:

• *A priori* definition of a balanced set of candidate reference genes, by applying additional physiological knowledge

• Testing of candidate reference gene stability using different algorithms, without using data of the investigated target genes

• Comparison of reference gene stability among groups of potential genetic differentiation

• Use of at least two reference genes which are stable among the group for which a statement is to be made

• Use of a real-time PCR quantification method that considers individual reaction efficiencies, accounting for variations among samples

• Partial sequencing and analysis of sequence similarity of target genes, to support comparability of physiological function

For comparing organismal responses to combined stressors, high-stress environments like intertidal zones can serve as natural laboratories [12-14]. The classic heat shock response (HSR) involving increased expression of heat shock proteins (Hsps) of the Hsp70 class [15] has been proposed as an integrative measure of the stress levels to which organisms are acutely exposed, and constitutive expression of Hsps may be a physiological prerequisite for organisms in ecological niches close to their specific tolerance limits [16-18]. Therefore, gene expression data of different Hsp isoforms can be highly useful to define thresholds for physiological function beyond which fitness and long-term species survival are impaired [19].

We conducted a set of field experiments with intertidal and sublittoral populations of limpets of the genus *Nacella*, examining their constitutive Hsp expression and their heat shock response to tidal emersion under natural habitat conditions. Samples were available that had been analyzed in a previous study of two *Nacella* species from the South American Magellan region (*N. magellanica, N. deaurata*), analyzing the heat shock response (HSR) on a shore level and a North–south geographical climate gradient [20]. Additionally, we used samples from the Antarctic congener *N. concinna* to expand the ecological and biogeographic gradient, now comprising polar to cold-temperate environments.

Intertidal and sublittoral *Nacella* specimens appear to be differentially adapted to tidal exposure and are morphologically distinguishable. While Magellanic *Nacella* have been divided into two main species corresponding to tidal zonation, *N. magellanica* and *N. deaurata*[21]*,* tidal groups of the more distant Antarctic congener *N. concinna* are considered different ecotypes of the same species [22]. As the degree of genetic isolation is still under debate in both cases, in this study, we treat all groups as ‘subpopulations’ under the hypothesis that their last common ancestor had to adapt to different local environmental conditions.

Using the data from air exposure experiments with five different *Nacella* limpet subpopulations, we investigate whether acute heat shock responses and constitutive Hsp70 expressions differ between habitats, to illustrate the dependence of these comparisons on methodological aspects and the insights that differences among populations can provide into the adaptation of populations to environmental parameters.

## Methods

### Sampling and experimental treatment

**Figure 1:**
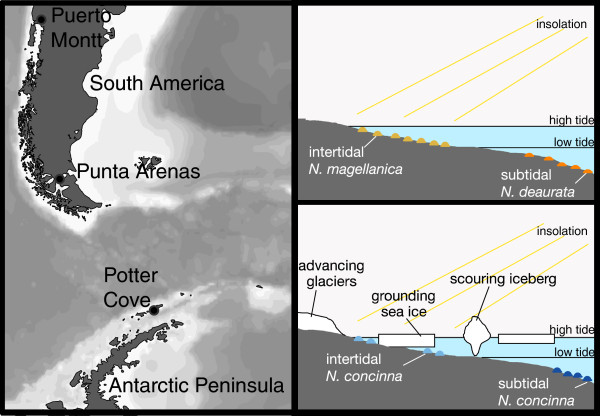
**Geographic locations and habitat characteristics of sampled subpopulations of *Nacella spp. ***Map of Southern South America and Antarctic Peninsula with sampling locations (left), and schematic overview of intertidal habitats and environmental stressors in the intertidal in South America (upper right) and Antarctica (lower right).

Sampling and experiments were performed at three locations under different environmental conditions (Figure 1; Tables 1 and 2). Sample collection of South American *Nacella* (intertidal *N. magellanica* and subtidal *N. deaurata*) was carried out at two different sites in Chile, control animals were dissected and snap frozen immediately, and air exposure experiments were carried out on site. Antarctic *N. concinna* were sampled in intertidal and sublittoral subpopulations at Potter Cove, King George Island, South Shetlands and acclimated submersed in aerated, 0°C cold seawater for 3 weeks before sampling and conducting air exposure experiments. See [20] and [23] for details on sampling and experimental setup.

**Table 1:** **Sampled subpopulations of *Nacella spp.*, local tidal emersion regimes and experimental conditions**

**Site**	**Coordinates**	**Species**	**Tidal zone, depth**	**Tidal emersion**	**N / group**	**Air T [°C]**	**Weather**
Puerto Montt (PM)	41°30^′^42^′^^′^S 73°00^′^55^′^^′^W	*N. magellanica*	Intertidal, 5–6 m	Spring tides	6	16–24	Sunny
Punta Arenas (PA)	52°56^′^56^′^^′^S 70°47^′^44^′^^′^W	*N. magellanica*	Intertidal, 1 m	2–4 h / d	6	9–17	Sunny/ cloudy
		*N. deaurata*	Sublittoral, 2 m	-	6		
Potter Cove (PC)	62°14^′^25^′^^′^S, 58°40^′^51^′^^′^W	*N. concinna*	Intertidal, 1 m	2–4 h / d	8-10	0	protected
			Sublittoral, 13–15 m	-	8-10		

N = number of sampled individuals of the subpopulations; air T = temperature range in the course of the air exposure experiments.

**Table 2:** Stability ranking of candidate reference gene expression by two different algorithms, calculated for sampled subpopulations

		**GeNorm**			**NormFinder**		
		**Rank**	**Gene**	**M**	**Rank**	**Gene**	**Stability**
Puerto Montt	*N. magellanica*, intertidal	1st + 2nd	**Grp78 + Actin**	1.163	1st	**Grp78**	0.38
		+3rd	HIF	1.316	2nd	**HIF**	0.63
					best pair	Grp78 + Actin	0.388
Punta Arenas	*N. magellanica*, intertidal	1st + 2nd	**Grp78 + HIF**	0.89	1st	**Grp78**	0.297
					2nd	**HIF**	0.494
		+3rd	H3	1.15	best pair	Grp + HIF	0.33
	*N. dearauta*, sublittoral	1st + 2nd	**Grp78 + HIF**	0.86	1st	**Grp78**	0.14
					2nd	**HIF**	0.29
		+3rd	Actin	1.06	best pair	Grp78 + HIF	0.163
Potter Cove	*N. concinna, *intertidal	1st + 2nd	**HIF + H3**	0.869	1st	**Grp78**	0.198
					2nd	**HIF**	0.211
		+3rd	Grp78	0.929	3rd	H3	0.285
					best pair	Grp78 + HIF	0.158
	*N. concinna,* sublittoral	1st + 2nd	**HIF + H3**	0.636	1st	**H3**	0.22
					2nd	**HIF**	0.227
		+3rd	Grp78	0.848	3rd	Grp78	0.3
					best pair	HIF + H3	0.169
All controls (0h)	*Nacella*	1st + 2nd	**Grp78 + Actin**	0.94	1st	**Grp78**	0.573
					2nd	**Actin**	0.62
		+3rd	H3	1.388	3rd	H3	0.704
					best pair	Grp78 + HIF	0.486

First three ranks are shown. GeNorm calculates an average expression stability M based on the standard deviation between all genes and samples, while NormFinder returns a model-based stability value between population/treatment groups.

Air temperatures during the air exposure experiments in Chile fluctuated between 9 and 24°C and were recorded in 30-min intervals throughout each experiment. In the Antarctic experiments temperature was kept constant at 0°C and animals were exposed in a glass container with air exchange. Samples from air exposure experiments were taken after 0 h (controls), 2, 6 and 12 h in South America and at 0, 2, 6, 12, and 24 h in the Antarctic. See Table 2 for details of experimental treatment conditions.

### Selection of reference genes & primer design

As candidate reference genes for normalization of Hsp70 expression values we evaluated β-actin, histone H3, GRP78 and HIF-1α. The choice of these candidate reference genes was based on availability of primer sequence information and *a priori* physiological considerations: (1) ß-actin is a cell skeleton protein commonly used as a reference gene in gene expression studies. Nevertheless, there is indication that it can be down-regulated under stressful conditions such as hypoxia, when growth comes to a halt and cell division is interrupted [11] (2) Histone H3 is a chromatin protein used for binding and packaging of DNA in the cell nucleus, and highly conserved among taxa [24] (3) GRP78 is a constitutive molecular chaperone of the Hsp70 class located in the endoplasmatic reticulum and involved in protein secretion [25]. GRP78 was reported to be relatively stable under short-term stress in *Nacella*[26] (4) Hypoxia Inducible Factor 1 subunit α (HIF-1α) is a central transcription factor for responses to low oxygen levels and reported to be constitutively transcribed. Regulation is, at least in mammals, achieved at the protein level [27,28].

The four genes are involved in unrelated physiological tasks, which decreases the probability of co-regulation.

Degenerate primers were used to amplify fragments of the target heat-shock proteins (Hsp70A, Hsp70B, Hsc70 and GRP78) and of β-actin in genomic DNA samples of *N. magellanica* and *N. deaurata*. PCR products were cloned and DNA sequences obtained from cloned fragments were checked for matching with already published primers. When mismatches were found in the cloned fragments, new primers were designed. For details on primer design, see [20].

### RNA extraction, real-time RT-qPCR and sequencing

Foot tissue samples were homogenized and total RNA extracted under RNase-free conditions, and RNA was reverse transcribed into cDNA using oligo-(dT)_18_ primers and a protocol adjusted for dissolving secondary structures, as detailed in [20].

Real-time qPCR was conducted as previously described [20], including negative -RT and NTC (no template control) controls in each run and distributing treatment groups evenly between runs. Primer concentrations were optimized, and amplification efficiency and linear range of the assay tested by relative standard curves, while specificity of the RT-qPCR amplification was confirmed by melt analysis of each single reaction and by sequencing samples for each product peak [20].

By partial sequencing of the three Hsp70 isoforms from four samples of each species, we obtained additional support for the high conservation and orthology of the Hsp70 isoforms in Nacellids, and thus for comparability of gene expression levels between the three different *Nacella* species. The 960 base-pair sequences represent 40 – 50% of the Hsp70 mRNA, as estimated by alignment with complete coding sequences of mollusc Hsp70s available in GenBank (Pöhlmann et al., in prep.). While the three Hsp70 isoforms exhibit an overall identity of 61%, Hsp70B and Hsc70 share 98% and 99% sequence identity, respectively, and Hsp70A is slightly more variable with 96.5% conservation between the three Nacellid species. Sequences of the three genes unambiguously contained the respective primer regions used for qPCR, confirming specificity of amplification in the assessment of gene expression levels.

### Data processing and statistics

To increase the reliability of qPCR data, we used the Second Derivative Maximum method for quantification, which is equivalent to the conventional Threshold Cycle (C_T_ or Cq) method [29,30], but does not assume equal reaction efficiencies among individual reactions. We regard this as important when comparing separate populations that potentially have different mutations in the amplified fragments, changing primer binding and reaction efficiency.

Relative quantities calculated by the ’Comparative Quantification’ feature in the Rotor-Gene Q Series software V. 1.7.94 (Qiagen) were transferred to Excel 2002 (Microsoft Corp.) and stability of candidate reference genes tested by the macros GeNorm [31] and NormFinder [32]. For normalized expression values, take-off points and individual amplification values were transferred to the REST 2009 software [33], where relative up- or down-regulations were calculated and tested for statistical significance by the integrated Bootstrap randomization test (2000 iterations) between 0h controls and samples for each subpopulation and treatment group.

## Results

### Reference gene stability

Candidate reference gene expression stability under the experimental treatment was calculated separately for the five subpopulations by the two algorithms (GeNorm and NormFinder). Grp78 and HIF-1 were identified as the two most stable genes by both algorithms for the Punta Arenas subpopulations. For the Puerto Montt population, GeNorm proposed Grp78 and *β*-actin, and NormFinder ranked Grp78 as the most stable and HIF-1 as the second most stable gene. For the *N. concinna* subpopulations of Potter Cove, GeNorm ranked HIF-1 and H3 as most stable genes, followed by Grp78, while NormFinder proposed Grp78 and HIF-1 for the intertidal subpopulation and HIF-1 and H3 for the sublittoral group (Tab. 2). On the basis of their overall consistent performance and robustness against differences in the detection algorithms, the combination GRP78 and HIF-1 was subsequently used as a reference for normalization of expression values under experimental treatment. For comparison of constitutive expression levels, values of untreated control samples were checked as one group, and the combination of GRP78 and β-actin used for normalization as suggested by both algorithms.

### Heat shock response

**Figure 2:**
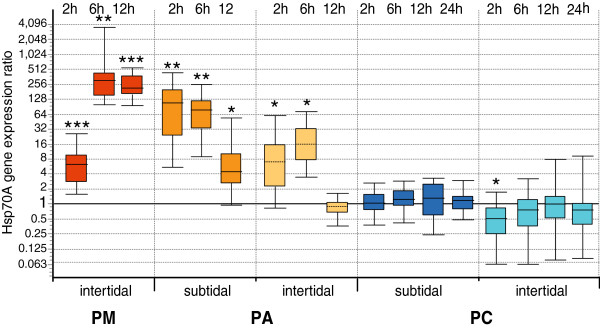
**Hsp70A gene expression in air exposed individuals from all subpopulations, relative to control animals. **Values normalized to reference genes Grp78 and HIF-1 for subpopulations in Puerto Montt (**PM**), Punta Arenas (**PA**) sublittoral and intertidal, Potter Cove (**PC**) sublittoral and intertidal. Asterisks indicate significant up- or down-regulation compared to the control animals (Bootstrap test by REST 2009, *p < 0.05, **p < 0.01, ***p < 0.001). Boxes represent 50% of values with median value (dotted line), whiskers are extreme values.

**Figure 3:**
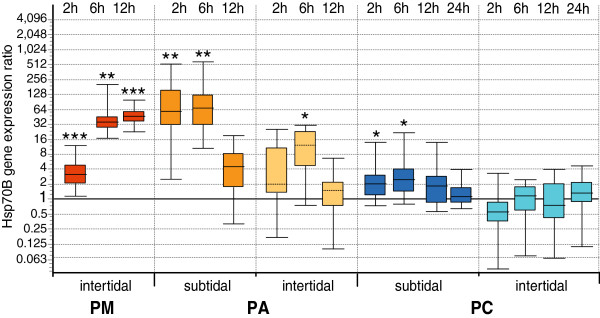
**Hsp70B gene expression in air exposed individuals from all subpopulations, relative to control animals. **Values normalized to reference genes Grp78 and HIF-1 for subpopulations in Puerto Montt (**PM**), Punta Arenas (**PA**) sublittoral and intertidal, Potter Cove (**PC**) sublittoral and intertidal. Asterisks indicate significant up-regulation compared to the control animals (Bootstrap test by REST 2009, *p < 0.05, **p < 0.01, ***p < 0.001). Boxes represent 50% of values with median value (dotted line), whiskers are extreme values.

After normalization to Grp78 and HIF-1, the stress responses in both isoforms, Hsp70A and Hsp70B, appeared very similar in timing, but differed markedly between the investigated subpopulations (Figures 2 and 3).

South American limpets mounted a stress response that involved up-regulation of inducible heat shock proteins by several orders of magnitude over control levels. In the Puerto Montt subpopulation, the highest average levels of all samples were reached at 6 h and 12 h following emersion, with 240 to 300-fold up-regulation of Hsp70A and 40 to 47-fold up-regulation of Hsp70B*.* In the Punta Arenas subpopulations, maximum expression levels were more variable at the individual level and recorded sooner after exposure to stress than in the PM subpopulation. Average levels of 87-fold (Hsp70A) and 70-fold (Hsp70B) occurred in sublittoral *N. deaurata* already in the 2 h treatment, while intertidal animals displayed comparatively moderate up-regulations of 4–16-fold in the 2 h and 6 h treatments (Hsp70A*)* and in the 6 h treatment (Hsp70B*)*.

In Antarctic *N. concinna,* a two-fold up-regulation occurred of Hsp70B in sublittoral individuals after 2 h and 6 h of air exposure. Air-exposed intertidal *N. concinna* displayed no significant deviation from controls in the average Hsp70B expression, but Hsp70A expression was conspicuously down-regulated to 50% in the 2 h treatment, with some individuals showing 16-fold less transcript levels for both Hsp genes compared to submerged controls.

### Constitutive Hsp70 levels

**Figure 4:**
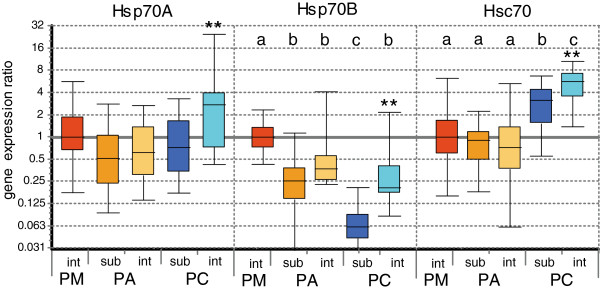
**Constitutive levels of gene expression for Hsp isoforms Hsp70A, Hsp70B and Hsc70 in *Nacella *subpopulations. **Values of control animals, relative to Puerto Montt subpopulation and normalized to reference genes *Grp78 *and *β-actin*, for subpopulations at Puerto Montt (**PM**), Punta Arenas (**PA**) sublittoral and intertidal, Potter Cove (**PC**) sublittoral and intertidal. Letters indicate significant differentiation among the subpopulations. Asterisks indicate significant up-regulation compared to sublittoral animals at the same site (Bootstrap test by REST 2009, **p < 0.01). Boxes represent 50% of values with median value (dotted line), whiskers are extreme values.

Expression values from control animals without air exposure were used to represent constitutive expression of heat shock proteins, and normalized to the most stable genes among the control animals, GRP78 and β-actin. Average constitutive expression was significantly different between subpopulations, with Puerto Montt animals displaying the highest constitutive levels of Hsp70B (Figure 4). Sublittoral *N. concinna* had the lowest constitutive expression of this inducible Hsp isoform, but both *N. concinna* subpopulations had higher constitutive levels of the heat shock cognate Hsc70 than their South American congeners. In the Antarctic limpets, unstressed chronic expression of all three Hsps was up to 4-fold elevated in intertidal animals above the levels reached by their sublittoral conspecifics.

## Discussion

### Reference gene assessment

We compared the expression of three Hsp70 isoforms (Hsp70A, Hsp70B, Hsc70) among different *Nacella* subpopulations across a biogeographic gradient of environmental conditions spanning from Northern Patagonia into Western Antarctica. Our objective was to assess the utility of the heat shock stress response in ecological comparisons of geographically distant subpopulations of a species complex with different climatic background. Therefore, we were concerned not to assume identical genetic predispositions and explored the methodological necessities for gaining comparable expression data. We used an *a priori* defined set of reference genes with different physiological tasks (β-actin, histone H3, Grp78 and HIF-1α) for the stability tests to minimize the risk of choosing co-regulated genes.

Both algorithms for identification of least strongly responding expression levels, GeNorm and Normfinder, produced very similar results, but disagreed in some points (cf. tab. 2). For the quantitative comparison between subpopulations, Grp78 and HIF-1 were used for normalization of heat shock gene transcription in all animals. For the intertidal and sublittoral subpopulations of *N. concinna*, in which slightly better stability values were calculated for other reference gene combinations, the difference was small and it was verified that all conclusions would also hold with the proposed optimal combination. For normalization of constitutive heat shock gene expression, the control animals were tested separately, and Grp78 and β-actin were rated as the most stable combination. The commonly used reference gene β-actin thus appeared as suitable for normalization of constitutive levels but not for inducible responses to acute stress in our study.

The established reference gene combinations may serve as a useful starting point for further stress experiments in *Nacella* or other limpets. However, our study demonstrates that in non-model organisms stability of reference genes cannot be derived from theory, but instead must be determined empirically from a carefully chosen set of candidate genes using different detection algorithms. The results show how gene expression patterns in limpets can vary depending on the adaptive history of the investigated population. Further, the selection of reference genes may certainly depend also on the nature of the investigated stressor.

### Comparison of the stress response among subpopulations

The sampled *Nacella* subpopulations showed different cellular stress responses and displayed different levels of constitutive Hsp gene expression under the respective temperature regimes.

We have previously described the differences in timing and intensity of the Hsp70 stress response in South American *Nacella*[20]. In this work, we improved data handling by extracting amplification values from raw fluorescence curves by the SDM method, which considers individual reaction efficiencies, and by a posterior statistical method that does not assume a normal distribution (bootstrapping vs. ANOVA), and obtained expression changes with higher levels of statistical significance among all subpopulations and treatments. All South American subpopulations have been able to mount a heat shock response, with the most clear-cut HSR observed in limpets near the northernmost limit of their distribution range (Puerto Montt). In contrast, the samples from Antarctic limpets at King-George Island show no significant induction of heat shock proteins compared to controls in most treatments. Instead, a down-regulation of Hsp70A was apparent in the 2 h treatment and in some exposed individuals of all other treatment groups, possibly indicating a general down-regulation of many genes including the Hsp70 genes. Nevertheless, when interpreting this result it has to be kept in mind that no statement on overall transcription levels or mRNA/rRNA ratio can be made with the RT-qPCR data, because of the normalization to overall RNA amounts performed before reverse transcription.

After normalization to the most stable genes among control animals, we can substantiate the differences between the investigated subpopulations by showing that Puerto Montt limpets also possess higher constitutive expression levels of the inducible isoform Hsp70B at the time of sampling than limpets at the colder Punta Arenas site, suggesting a connection of constitutive expression of heat shock proteins with water temperatures in the habitat. Also, intertidal *N. concinna* in our study maintained higher control levels of Hsp70A, Hsp70B and Hsc70 than the sublittoral group, even after an acclimation period. This discovery suggests an increased constitutive expression triggered on a long-term basis by regular air exposure or fixed by genetic adaptation. This result is interesting when juxtaposed with genetic data, because no genetic distinction between intertidal and sublittoral *N. concinna* has been found so far and they are suspected to form a single genetic entity [22].

The comparison of the induced and constitutive levels of all Hsp70 isoforms between the subpopulations can be used to indicate possible connections to environmental factors. Comparing the HSR among all subpopulations, the up-regulation levels of heat shock proteins correlate with the air temperature regime experienced by the animals upon tidal exposure. Among South American limpets, *N. magellanica* from Puerto Montt employ extreme heat shock responses under air exposure at 16 – 24°C, while *N. magellanica* and *N. deaurata* from Punta Arenas employ less strong heat shock responses at an air temperature of 9 – 17°C. Antarctic *N. concinna* show on average only a very limited up-regulation of Hsp70B after 2 h and 6 h under the constant temperature regime at 0°C.

While we did not find a pronounced HSR in *N. concinna*, we detected increased control levels of the heat shock cognate Hsc70 in both the intertidal and the sublittoral ecotypes compared to *N. magellanica* and *N. deaurata* under higher sampling temperatures. Constitutive expression of heat shock proteins has been suspected to be a feature of adaptation to the polar environment for some Antarctic fish [34], and elevated expression of constitutive Hsps in the intertidal group has been reported for freshly sampled *N. concinna*[35]. The interpretation of constitutive heat shock protein expression as an adaptation strategy to cold environments can gain additional support by studies comparing gene expression among populations, when methodological comparability is ensured. The subpopulation comparison might indicate that Antarctic intertidal *Nacella* employ a different physiological strategy to endure life in the polar intertidal zone compared to their congeners in more temperate climates, based on constitutive production of Hsc70 and, possibly, metabolic reduction upon exposure, instead of a pronounced heat shock response.

These results provide insights into the diverse roles that Hsps can have in adaptation to different kinds of physiological stress in an environmental context. While short-term up-regulation of some Hsp70 isoforms represent a mechanism of adaptation to tidal exposure under high air temperatures that can be extremely important in terms of transcription levels, other isoforms can undergo a long-term adaptation in constitutive expression levels in a cold environment. These two mechanisms represent potential evolutionary limitations of *Nacella* near either extreme of its biogeographic distribution. The population at Puerto Montt inhabits the northern limit of the *Nacella* distribution range, and the physiologically possible strength of the HSR in response to air exposure, supported by constitutive production of Hsp70B, are putative limiting factors in colonization of warmer intertidal habitats. On the other hand, *N. concinna* on the Antarctic peninsula is the only limpet living in a polar habitat after the last glacial maximum, which may require the constitutive production of Hsc70 as a response to cold stress.

Some differences in the experimental protocols may limit the ecological meaningfulness of the differences in stress responses between South American and Antarctic populations in our study, because at the time of conducting the experiments a quantitative comparison was not planned. Nevertheless, the methodological suggestions with regard to gene expression measurements exemplified in the present work should prepare the grounds for a reliable quantitative comparison of physiological responses of populations in future works. By conducting experiments under identical temperature regimes with limpets of all populations, it will be possible to test whether the observed differences are caused by phenotypic plasticity or partially represent intrinsically fixed adaptations of the populations to their environment. By integrating other protective mechanisms, including morphological, behavioural and other physiological adaptations, it will be possible to advance towards a comprehensive understanding of the effects of environmental stress on organisms under different environmental conditions.

## Conclusions

The gene expression data from *Nacella* were used for a case study to explore the methodological prerequisites of stress response comparisons over biogeographic distances. Restrictions and implicit assumptions in gene expression quantification methods have to be kept in mind when performing comparative physiology studies under potential genetic divergence. Adopting data normalization practices from biomedical qPCR studies and comparing reference gene expression stability between subpopulations, a set of reference genes can be identified that not only serves to minimize variation, but yields valuable additional information to create a more reliable basis for ecological comparison between all sampled populations.

This case study can serve as a starting point to establish a normalization framework for the use of gene expression data in comparative physiology studies and gives hints for the use of the cellular stress response as a biomarker to study environmental stress and stress adaptation under changing environmental conditions. To gain a complete picture, it will be important which Hsp70 isoforms are chosen for comparison and constitutive expressions have to be compared with acute stress responses. When methodological criteria for comparability are established, Hsp70 or other characteristic stress response genes measured by RT-qPCR and / or microarrays can provide fast and inexpensive parameters for comparing environmental stress along ecological gradients. This will help to establish and model the mechanistic links between environmental change and adaptive responses of organisms.

## Abbreviations

CT: Threshold cycle; CP: Crossing point; Grp78: Glucose-regulated Protein (78kDa molecular chaperone); H3: Histone H3a; HIF-1: Hypoxia-inducible Factor 1; Hsc70: Heat-shock cognate (70kDa molecular chaperone); Hsp70: Heat-shock protein (molecular chaperone of the 70kDa class); HSR: Heat shock response; mRNA: Messenger ribonucleic acid; PA: Punta Arenas (experimental site located in the Strait of Magellan); PM: Puerto Montt (experimental site located in Northern Patagonia); PC: Potter Cove (experimental site located on the Antarctic Peninsula); qPCR: Quantitative (real-time) polymerase chain reaction; RNA: Ribonuleic acid; RT: Reverse transcription; Tm: Melting temperature.

## Competing interests

The authors declare that they have no competing interests.

## Authors’ contributions

SK carried out molecular genetic measurements and data analyses, developed the normalization concept and drafted the manuscript. KP performed sampling and experimental treatments, and participated in the design of the study and the interpretation of the results. CH and DA designed and coordinated the study and guided the interpretation of the results. All authors read and approved the final manuscript.
